# Clinical and radiological evaluation of radial neck factures in children and adolescents treated by percutaneous leverage reduction with Kirschner wire stabilization

**DOI:** 10.1007/s00264-023-05965-w

**Published:** 2023-09-09

**Authors:** Norbert Guzikiewicz, Krzysztof Małecki, Marcin Sibiński, Kryspin Niedzielski

**Affiliations:** 1 Clinic of Orthopaedic and Traumatology, Polish Mother’s Memorial Hospital Research Institute, Lodz, Poland; 2 Clinic of Orthopaedics and Paediatric Orthopaedics, Medical University of Lodz, 92-213 ul, Pomorska, 251 Łódź, Poland

**Keywords:** Fracture of the proximal radial metaphysis, K-wire stabilization, Operative treatment, Percutaneous leverage, Proximal radius

## Abstract

**Purpose:**

The aim of the study was to evaluate the clinical and radiological results of surgical treatment of radial neck fractures in children and adolescents by percutaneous leverage with Kirschner wire stabilization.

**Methods:**

A retrospective clinical and radiographical evaluation was performed on a cohort of 61 patients (mean age 9.7 years; range 3 to 15) with isolated, unilateral radial neck fractures treated between 2009 and 2019. The mean duration of follow-up was 4.2 years (range 2 to 9 years). All fractures were types III and IV according to Judet’s classification.

**Results:**

After mean follow-up, the radiographic results according to Metaizeau were rated as excellent in 70.5% of respondents, good in 27.9%, satisfactory in 1.6%. According to Mayo Elbow Performance Score, 95.1% of respondents obtained a very good result, 3.3% good, and 1.6% satisfactory. The mean radial neck–shaft angle changed from a mean 51.5° before operation to 3.8° postoperatively (*p*<0.001). The mean translation was 3.1mm before surgery and 0.5mm postoperatively (*p*<0.001). No limb axis deviation, elbow joint instability, and infection of the implant insertion site were observed. No statistically significant differences were noted between girls and boys (*p*>0.05).

**Conclusions:**

Our findings indicate that percutaneous leverage with Kirschner wire stabilization is an effective and safe method for treating isolated radial neck fractures, characterized by a low risk of iatrogenic complications.

## Introduction

Radial neck fractures in children are rare, only accounting for about 1% of all fractures. They can be isolated or accompany other injuries of the elbow joint. In children, the approach to treatment remains controversial; however, non-displaced fractures, or those with minor displacements, are treated with immobilization, while types III and IV according to Judet, require surgery [1]. Multiple possible techniques have been described. Possible surgical treatments include closed reduction without internal fixation, closed reduction with intramedullary fixation, combined methods (i.e., percutaneous leverage technique accompanied by intramedullary fixation), or open reposition with internal stabilization [2–5]. Of these, percutaneous leverage reduction and fixation using Kirschner wires is the approach favoured in the institution of the first author. The aim of treatment is to minimize the adverse consequences of injury and surgical intervention and to achieve good function of the elbow joint.

The aim of the study was to evaluate the clinical and radiological results of surgical treatment of radial neck fractures in children and adolescents by percutaneous leverage with Kirschner wire stabilization.

## Material and methods

A retrospective evaluation was performed of a group of patients who had previously been treated surgically for isolated, unilateral radial neck fractures by percutaneous leverage reduction with Kirschner wire stabilization. In total, 61 children were studied, 36 girls (59.0%) and 25 boys (41.0%); all had received treatment in the years 2009–2019. In all subjects, the proximal growth cartilage of the radius was open at the time of injury. Surgery was performed on average 2.0 days after injury (range 0 to 12 days). The mean age of the subjects at the time of injury was 9.7 years (range 3 to 15). The mean age at the last check-up was 13.7 years. The mean duration of follow-up was 4.2 years (range 2 to 9). The displacement of fragments was > 30° measured by radial neck–shaft angle and/or >2 mm translation.

The following patients were excluded from the study: (1) people with concomitant ulnar fractures, (2) with concomitant dislocation of the elbow joint, (3) with closed growth cartilage of the radial head at the time of injury, (4) after an unsuccessful attempt at surgical treatment by another method.

The subjects were evaluated clinically and radiologically on the basis of X-ray of the elbow in AP and lateral views. The post-traumatic radiological assessment was based on the Judet classification [1] (Table 1). The radial neck–shaft angle was determined as the angle between the line perpendicular to the articular surface of the radial head and the axis of the radius. Translation was determined as the distance in millimeters measured from the center of the radial head to the axis of the radius. After surgery, the Metaizeau scale [4] (Table 2) was used for radiological evaluation of elbow X-ray, while the Mayo Elbow Performance Score (MEPS) was used for clinical evaluation. MEPS is a 100 points scale consisting of four subscales: pain (maximum 45 points), ROM (maximum 20 points), stability (maximum 10 points) and daily function (maximum 25 points) [6].

**Table 1:** Judet classification of radial neck fractures, along with the number of patients in groups

Judet classification		Number of patients (%)
Type	Degree of displacement	
I	No displacement or horizontal shift of epiphysis	0
II	< 30° angulation	0
III	30° a 60° angulation	43 (70.5%)
IVa	60° a 80° angulation	
IVb	> 80° angulation	18 (29.5%)

**Table 2:** Radiological Metaizeau classification after surgical treatment, along with the number of patients in groups

Metaizeau classification		
Result	Reduction achieved	Number of patients (%)
Very good	Anatomic reduction	43 (70.49%)
Good	<20°	17 (27.87%)
Satisfactory	20–40°	1 (1.64%)
Poor	>40°	0 (0%)

### Operative technique and postoperative care

All patients were operated upon under general anaesthesia in a supine position. The operated upper limb was placed on a table with shoulder adduction and the elbow joint in extension. After preparing the operating field, using intraoperative fluoroscopy, the forearm was rotated from pronation to supination to find the greatest angular displacement of the head relative to the shaft. Following this, a 1.8-mm Kirschner wire was inserted percutaneously, slightly proximal into the fracture, parallel to the plane of the radial head, and then introduced into the fracture gap. The above method removes tissue tension during the leverage maneuver. By levering the Kirschner’s wire with the elbow in the various position, the radial head was reduced. Using a drill, the Kirschner wire was anchored in the opposite cortical layer of the radial epiphysis, increasing the stability of the implant and fracture. During the maneuver, no damage occurred to the joint surface. The fracture position and implant placement were then evaluated using intraoperative fluoroscopy. The implant was shortened to a length that allowed it to be enclosed by the skin, minimizing the risk of infection.

**Fig. 1:**
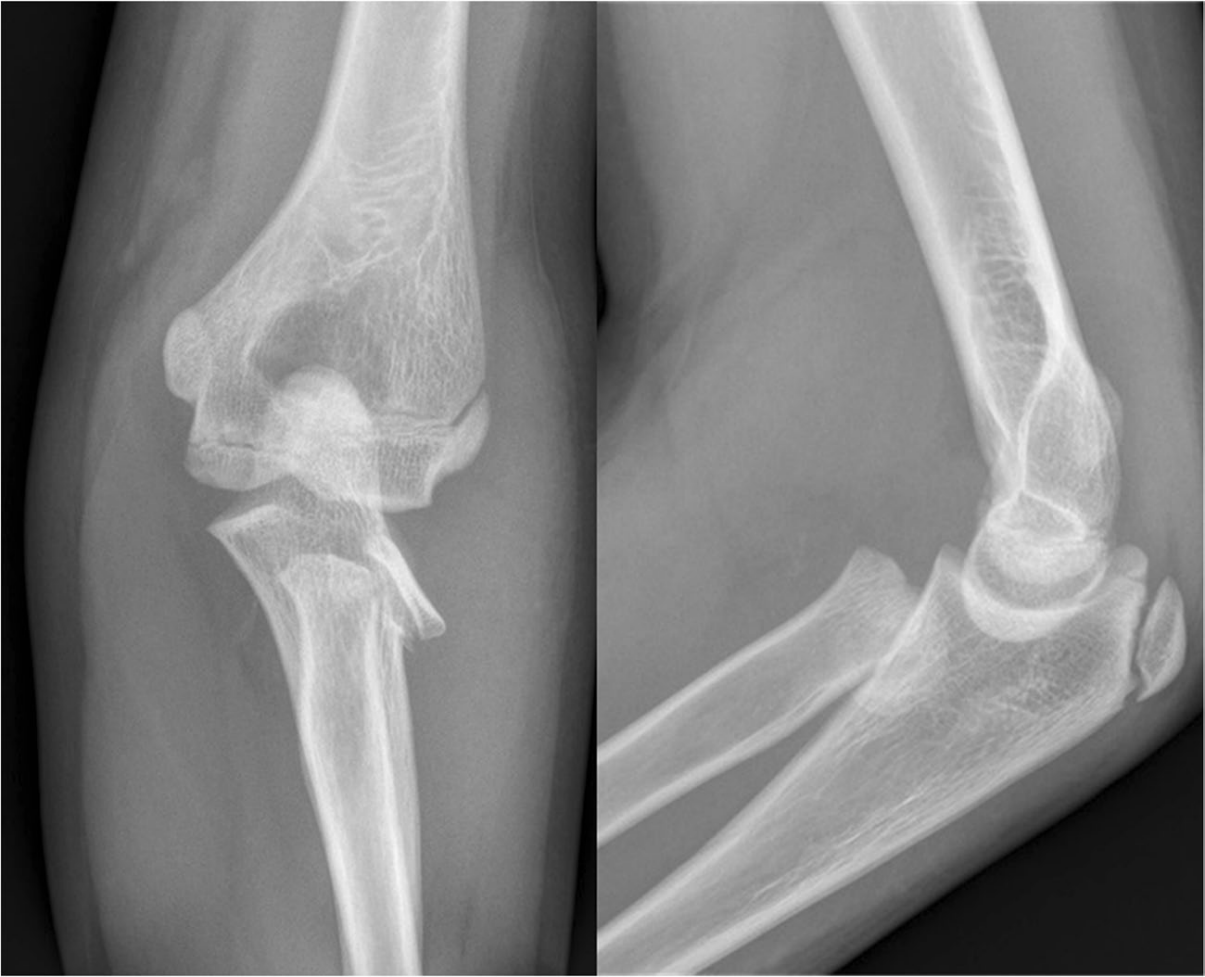
AP and L view radiograph of the elbow with radial neck fracture in a skeletally immature male

**Fig. 2:**
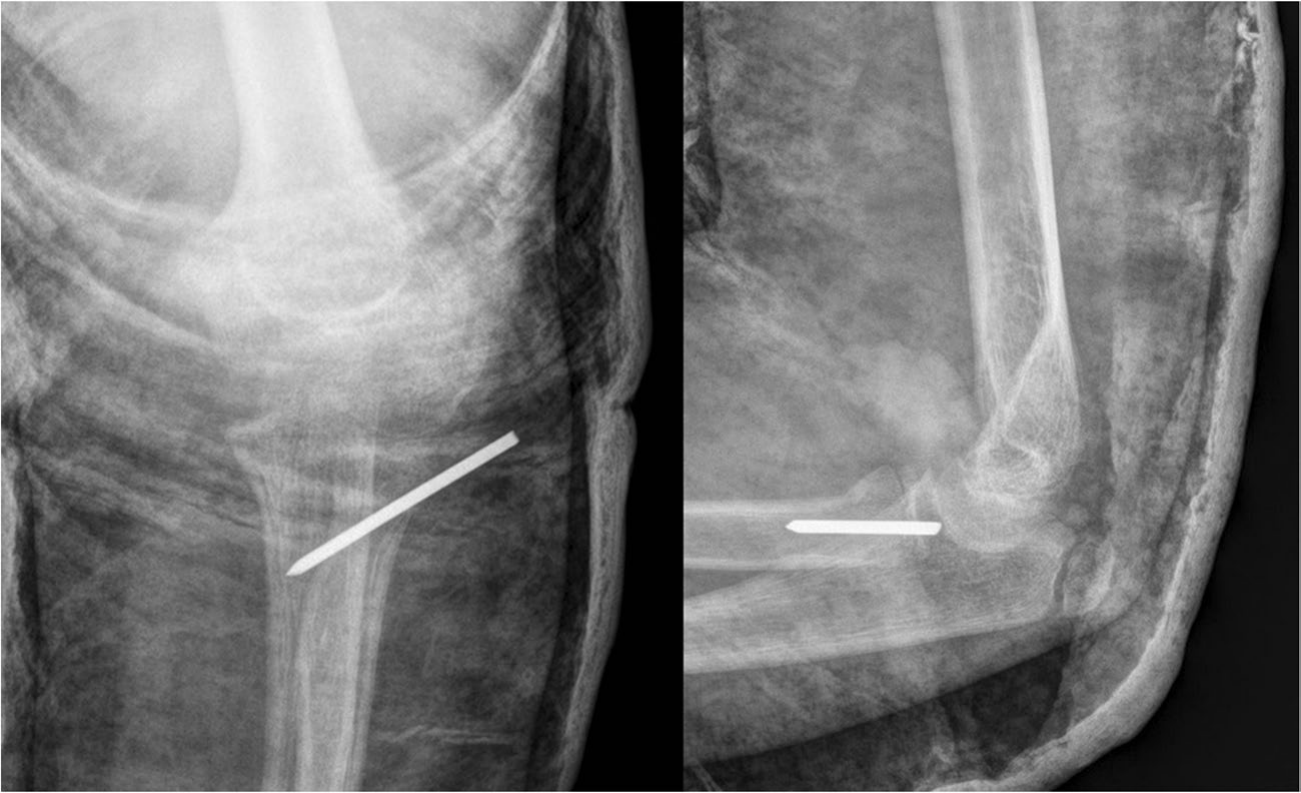
AP and L view radiograph of the elbow after surgical treatment by percutaneous leverage with Kirschner wire stabilization

**Fig. 3:**
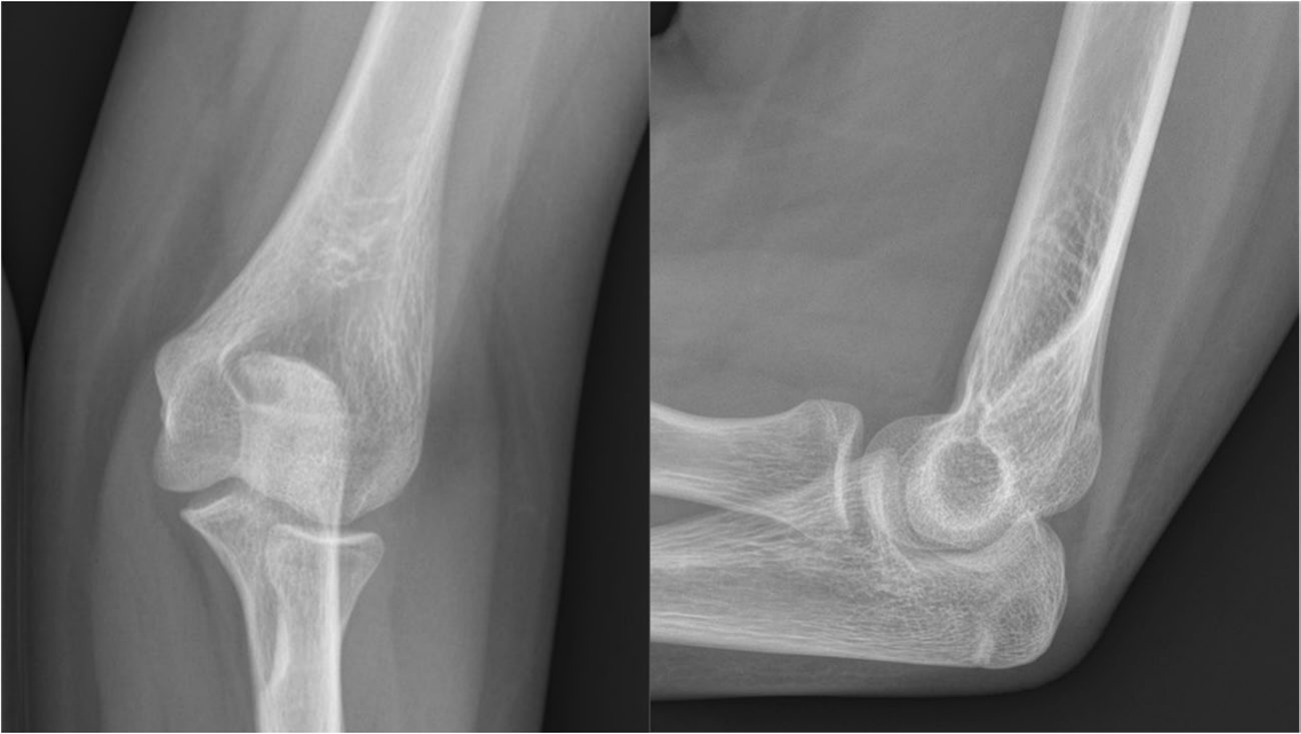
AP and L view radiograph of the elbow: final result, four years after surgical treatment

Only the leveraged technique using a single Kirschner wire was performed, without the use of any forced manipulations or unnecessary multiple insertions of the implant through soft tissues and fracture site. In each case, a full, long arm cast was applied on the intermediate position of the forearm at 90° of elbow joint flexion. Postoperatively, immobilization was maintained for a period of five weeks, following this, the cast was removed. X-ray of the elbow joint was performed. The implant was removed under local anaesthesia on an outpatient basis. Subsequently, initial rehabilitation was implemented. Figure 1 shows the elbow of an injured patient qualified for treatment using the leverage technique. Figure 2 shows the elbow after surgical treatment with plaster immobilization, and Figure 3, the final radiological result.

The study was approved by Bioethical Committee our Institute (project number 91/2018 /X/23) and was performed in line with the principles of the Declaration of Helsinki. Informed consent was obtained from a parent and/or legal guardian of all participants included in the study. If patients were 13 years old or more, the informed consent was obtained from both the participants and their parent and/or legal guardians, as required by law in our country.

### Statistical analysis

Numerical variables were described using central tendency measures, i.e., their weighted arithmetic mean and median, along with measures of dispersion, i.e., standard deviation, 95% confidence interval, and minimum-to-maximum values. Non-measurable (categorical) variables were represented as integer and percentage values.

Skewness and kurtosis was calculated to assess the normality of distribution, Levene’s test was used to confirm the homogeneity of variance. For normally distributed variables, the significance of any differences before and after surgery was confirmed by multifactor analysis of variance (ANOVA) with repeated measures. For non-normally distributed traits, any differences were compared using generalized estimating equations (GEE) with robust standard errors. All the multivariate models were controlled for age and sex.

A level of *p* < 0.05 was deemed statistically significant. All the statistical calculations were performed using IBM SPSS® Statistics, v. 28 (IBM® Corporation, Armonk, NY, USA). The statistical power of the tests performed amounted to 99%.

## Results

In the radiological assessment of X-ray after injury, 43 subjects were classified as type III according to Judet’s classification (70.5%) with a mean radial neck–shaft angle of 42.4°. The remaining 18 subjects (29.5%) were classified as type IV, with a mean radial neck–shaft angle of 70.6° (Table 1). For the whole study group, the mean radial neck–shaft angle was 51.5° after injury (range 35 to 90°) and 3.8° postoperatively (range 0 to 20°); the difference between these angles was statistically significant (*p*<0.001). The mean translation was 3.1mm before surgery (range 1 to 6mm) and 0.5mm postoperatively (range 0 to 2); this difference was also statistically significant (*p*<0.001) (Table 3).

**Table 3:** Descriptive statistics of the radial neck–shaft angle and translation before surgery, after surgery and during the last follow-up visit, with statistical analysis

Radiographic parameter	Phase of the study	Statistical values					*P* value
		M	Me	SD	95% CI	Min–max	
Radial neck–shaft angle (°)	Preoperative	51.5	45	14.4	45.8–55.2	35–90	< 0.001 a
	Postoperative	3.8	5	4.4	2.71–5.0	0–20	< 0.001
	Last follow-up	0.3	0	2.6	n/o	0–20	b
Translation (mm)	Preoperative	3.1	3	1.2	2.8–3.4	1–6	< 0.001
	Postoperative	0.5	0	0.6	0.3–0.6	0–2	a
	Last follow-up	0.03	0	0.3	n/o	0–2	< 0.001 b

^a^Statistical significance of the difference in preoperative and postoperative parameters; ^b^statistical significance of the difference in postoperative and last follow-up parameters; *M* mean; *ME* median; *SD* standard deviation; *CI* confidence interval; *min–max* minimum-to-maximum

In the postoperative X-ray of the elbow, an excellent result according to the Metaizeau classification was obtained in 43 subjects (70.5%), good in 17 (27.8%), and satisfactory in 1 (1.6%) (Table 2).

The assessment of elbow joint function (according to the MEPS) found 58 respondents (95.1%) to obtain a very good result, two (3.3%) good, and one (1.6%) satisfactory. In the scoring assessment, 55 patients obtained the maximum number of 100 points, three patients achieved 95 points, and of the three remaining patients, one each achieved 85 points, 80 points and 70 points. The mean MEPS was 98.7.

Table 4 presents the results of the assessment of the ranges of motion of the elbow and forearm of operated and non-operated limbs (i.e., without injury). No significant differences in elbow extension were observed between the two sides; however, the operated elbow demonstrated a statistically significant limitation in forearm rotation and flexion compared to the healthy side (Table 4).

**Table 4:** Descriptive statistics of the ranges of motion of the elbow joint of the operated and non-operated limb, along with statistical analysis

ROM	Operated/unoperated upper limb	Statistical parameter					*P* value
		M	Me	SD	95% CI	Min–max	
Flection (°)	Operated	138.9	140	3.3	138.0–139.7	130–145	0.005
	Unoperated	140.1	140	0.6	139.9–140.3	140–145	
Extension (°)	Operated	0.4	0	1.7	0.0–0.8	0–10	>0.05
	Unoperated	0.0	0	0.0	n/o	0–0	
Supination (°)	Operated	81.2	85	11.3	78.3–84.0	10–90	<0.001
	Unoperated	84.9	85	4.1	83.9–86.0	70–90	
Pronation (°)	Operated	82.1	85	11.3	79.2–85.0	10–90	0.035
	Unoperated	85.2	85	4.1	84.1–86.2	70–90	

*M* Mean; *ME* median; *SD* standard deviation; *CI* confidence interval; *min–max* minimum-to-maximum

Table 5 presents a more detailed analysis of the MEPS score among the subjects. No statistically significant differences in the examined parameters were noted between girls and boys (Table 5).

**Table 5:** Descriptive statistics of detailed MEPS scoring after surgery among subjects in general and by sex

MEPS subscale	Sex	Statistical parameter					*P* value
		M	Me	SD	95% CI	Min–max	
Pain	♀	44.6	45	2.5	43.7-45.0	30–45	0.794
	♂	44.4	45	3.0	43.2-45.0	30–45	
	All	44.5	45	2.7	43.8-45.0	30–45	
ROM	♀	10.0	10	0.0	n/o	10–10	0.999
	♂	10.0	10	0.0	n/o	10–10	
	All	10.0	10	0.0	n/o	10–10	
Stability	♀	24.4	25	2.0	23.8–25.0	15–25	0.639
	♂	24.2	25	2.4	23.2–25.0	15–25	
	All	24.3	25	2.1	23.8–24.9	15–25	
Everyday activities	♀	19.9	20	0.8	19.6–20.0	15–20	0.794
	♂	19.4	20	1.0	19.4–20.0	15–20	
	All	19.8	20	0.9	19.6–20.0	15–20	
Total	♀	98.9	100	5.1	97.2–100.0	70–100	0.629
	♂	98.4	100	4.9	96.4–100.0	80–100	
	All	98.7	100	5.0	97.4–100.0	70–100	

*M* Mean; *ME* median; *SD* standard deviation; *CI* confidence interval; *min–max* minimum-to-maximum

The group included one case of asymptomatic radial head hypertrophy (1.5%). Symptoms of transient posterior interosseous nerve palsy occurred in two cases (3.3%). Premature post-traumatic asymptomatic closure of growth cartilage was observed in 11 subjects (18.0%). In all patients, a growth plate was observed during injury (100%). In the follow up examination, all participants demonstrated a symmetrical axis for the elbow joints (100%) and full stability of the elbow joint (100%).

## Discussion

Although radial neck fractures may be quite rare in children, if incorrectly treated, they can lead to serious loss of function of the elbow joint and the entire upper limb. In about 50% of cases, proximal metaphyseal fractures of the radius are accompanied by other injuries, the most common of which are dislocation of the elbow joint, fracture of the olecranon and fracture of the coronoid process of the ulna. Treatment remains a challenge, starting from diagnosis through qualification for the procedure, and ending with the selection of the type and extent of the surgical technique. The main consideration when planning treatment is undoubtedly the degree of displacement, i.e., the radial neck–shaft angle and translation. Berstein et al. indicate good results in displacements < 60° in children under six years, due to the high potential for bone remodeling, while displacements >30° should not be left untreated in children over 12 years of age due to insufficient potential for remodeling [5]. Metaizeau et al. report complete bone remodeling in angular displacements between 20 and 30° in children under 12 years of age, while displacements 10–15° may not be remodeled in children over 12 years [6]. Possible treatments include long arm-cast immobilization, closed manual reposition, instrumental reposition, closed reposition with intramedullary stabilization, or open reposition with internal stabilization [2–5].

Although radial neck fractures in children after reposition appear to be stable, Futami et al. observed loss of reduction in 50% of cases (type IV according to Judet) treated by leverage without stabilization [7]. Similarly, Steinberg et al. observed secondary displacement of fracture fragments in 22 of 28 subjects following closed reduction without fracture stabilization [8]. Therefore, in the case of displaced unstable fractures, internal stabilization can prevent secondary displacement of fragments and the need for repeated intervention [5, 9, 10].

In the hospital of the first author, children and adolescents with displacement >30° of the radial neck–shaft angle and/or >2 mm translation, after previously unsuccessful manual reposition, are treated with surgery. Open reduction with internal stabilization is often associated with unsatisfactory results, and a fairly high incidence of complications in the form of limited range of motion of the elbow and limb function. As such, there is a great need to identify alternative surgical techniques for treating significant angular displacements with a lower risk of complications. In a review of open reduction with internal stabilization, Kumar et al. propose that the procedure should only be used in cases where other possible non-surgical and surgical methods have failed [9]. It is believed that the angular displacement of the radial head relative to the axis of the radius after surgical intervention should not exceed 20° [5, 10, 11].

In response to the increased risk of complications and poor elbow joint function associated with open reduction of radial neck fractures, it has been proposed is to minimize the risk of such complications by using reposition and closed stabilization methods, without opening the humero-radial joint [2–4, 10, 11]. The most widely used, simplest, and possibly most effective, approach is the Metaizeau technique. According to the author of the method, it is possible to obtain satisfactory repositioning in 75% of cases [4, 10]. However, fracture reposition problems may occur during significant angular displacements or fraction translation using the Metaizeau technique. In such cases, combined methods, e.g., percutaneous reposition by leverage or the joystick technique, may be helpful [12]. For example, Klitscher et al. reports using a combined method in six out of 28 surgical treatments due to problems with repositioning the fracture using the Metaizeau method alone [12].

Taking into account the anatomy of the retrograde vascular supply of the radial head, percutaneous leverage is presented as a risky approach which may increase the risk of impaired blood supply to the proximal radius [13]. However, no complications in the form of AVN of the radial head were observed in available studies evaluating the results of treatment using isolated percutaneous leverage or combined percutaneous leverage and Metaizeau [8, 14–19], which is in agreement with our present observations.

Similarly, concerns about damage to the posterior interosseous nerve using percutaneous leverage do not contradict the results of such treatment. No cases of permanent posterior interosseous nerve paresis were observed in available studies [8, 14–18]. In our present series, only two patients reported temporary posterior interosseous nerve palsy.

An innovative approach to the treatment of displaced radial neck fractures in children was proposed by Jong Yun Kim et al., who employed direct arthroscopic visualization of the reposition and Kirschner wire stabilization with good effect [19]. In addition, in line with ALARA principles (i.e., as low as reasonably achievable), i.e., minimizing exposure to X-rays, Lee et al. report good outcomes for patients treated with percutaneous leverage with Kirschner wire stabilization based on intraoperative ultrasound imaging [20].

The wealth of available surgical treatments, and the ongoing search for new ones, not only results from the diversity of potential injury, but also the lack of a single “best” and standardized way of treating them. The choice of therapy should take into account a range of factors, including the degree of displacement of the fracture, the accompanying damage to the elbow joint and forearm, the age of the patient, the time since the injury, the availability of surgical tools, and the experience of the surgeon.

The literature regarding the treatment of radial neck fracture by percutaneous leverage reduction and fixation in children is rather scare, and characterized by a variety of potential surgical modifications [5, 14, 16, 17]. Most research concentrates on new methods, mostly Metaizeau or combined methods [4, 10–12, 15]. The present article describes a relatively large homogenous group of types III and IV fractures according to Judet, all of while being isolated injuries. The presented operative technique is simple, easy to perform, effective and has a low complication rate with minimal injury to soft tissue. K-wire used is cheaper than other implants and offers good stability between fragments with a low risk of secondary displacement; in addition the K-wire can be removed in an ambulatory setting without any need for anesthesia. As such, this approach to performing percutaneous leverage reduction and fixation merits further interest.

## Conclusions

Our findings indicate that percutaneous leverage with Kirschner wire stabilization is an effective and safe method for treating isolated radial neck fractures, characterized by a low risk of iatrogenic complications.
